# Altered functional connectivity of the default mode network by glucose loading in young, healthy participants

**DOI:** 10.1186/s12868-018-0433-0

**Published:** 2018-05-31

**Authors:** Kenji Ishibashi, Keita Sakurai, Keigo Shimoji, Aya M. Tokumaru, Kenji Ishii

**Affiliations:** 1 Research Team for Neuroimaging, Tokyo Metropolitan Institute of Gerontology, 35-2 Sakae-cho, Itabashi-ku, Tokyo, 173-0015 Japan; 2 Department of Diagnostic Radiology, Tokyo Metropolitan Geriatric Hospital, 35-2 Sakae-cho, Itabashi-ku, Tokyo, 173-0015 Japan

**Keywords:** Resting-state functional MRI, Default mode network, Glucose, Precuneus, Posterior cingulate

## Abstract

**Background:**

The functional connectivity of the default mode network (DMN) decreases in patients with Alzheimer’s disease (AD) as well as in patients with type 2 diabetes mellitus (T2DM). Altered functional connectivity of the DMN is associated with cognitive impairment. T2DM is a known cause of cognitive dysfunction and dementia in the elderly, and studies have established that T2DM is a risk factor for AD. In addition, recent studies with positron emission tomography demonstrated that increased plasma glucose levels decrease neuronal activity, especially in the precuneus/posterior cingulate cortex (PC/PCC), which is the functional core of the DMN. These findings prompt the question of how increased plasma glucose levels decrease neuronal activity in the PC/PCC. Given the association among DMN, AD, and T2DM, we hypothesized that increased plasma glucose levels decrease the DMN functional connectivity, thus possibly reducing PC/PCC neuronal activity. We conducted this study to test this hypothesis.

**Results:**

Twelve young, healthy participants without T2DM and insulin resistance were enrolled in this study. Each participant underwent resting-state functional magnetic resonance imaging in both fasting and glucose loading conditions to evaluate the DMN functional connectivity. The results showed that the DMN functional connectivity in the PC/PCC was significantly lower in the glucose loading condition than in the fasting condition (*P* = 0.014).

**Conclusions:**

Together with previous findings, the present results suggest that decreased functional connectivity of the DMN is possibly responsible for reduced PC/PCC neuronal activity in healthy individuals with increased plasma glucose levels.

## Background

The default mode network (DMN), one of the resting-state brain networks, is characterized by hyperactivity when the brain is not engaged in specific behavioral tasks and low activity when the brain is focused on the external environment [1]. While performing various active tasks including novel, non-self-referential, and goal-directed tasks, the functional connectivity of the DMN consistently decreases [2, 3]. Although the mechanisms are not completely known, the DMN plays an important role in regulating complex cognition and behavior [4–6]. Its functional connectivity is impaired in patients with Alzheimer’s disease (AD) [7], and impairment worsens with disease progression [8]. Furthermore, altered functional connectivity of the DMN is associated with cognitive decline [9, 10]. Interestingly, the DMN functional connectivity can also decrease in patients with type 2 diabetes mellitus (T2DM) [11–13]. Although T2DM, characterized by insulin resistance and increased plasma glucose levels, is intuitively far from AD pathophysiology, T2DM is reportedly associated with cognitive decline and is a risk factor for AD [14]. Although it is unclear why T2DM is a risk factor for AD, the shared vulnerability of the DMN in the two diseases may reveal a functional association between them.

The functional connectivity in resting-state brain networks is measured by detecting spontaneous fluctuations in the blood-oxygen-level-dependent (BOLD) signals with functional magnetic resonance imaging (fMRI) [15]. Positive BOLD signals are presumably caused by altered cerebral blood flow, an index of neuronal activity [16]. Therefore, the functional connectivity in resting-state brain networks may be associated with neuronal activity [17]. As one of the most fundamental resting-state brain networks, the DMN comprises a set of interconnected brain regions, such as the precuneus/posterior cingulate cortex (PC/PCC), medial prefrontal cortex (MPFC), and lateral parietotemporal cortex (LPTC), with the PC/PCC being the functional core of the DMN [4, 5]. In AD patients, functional connectivity of the DMN is impaired [7, 8]; glucose metabolism, another index of neuronal activity, is compromised primarily in the PC/PCC [18, 19]. Therefore, in AD patients, decreased functional connectivity of the DMN is possibly associated with reduced neuronal activity in the PC/PCC.

Resting-state glucose metabolism, measured by fluorine-18-labeled fluorodeoxyglucose (^18^F-FDG) PET, is physiologically associated with neuronal activity [20]. Interestingly, recent studies using ^18^F-FDG PET showed that neuronal activity in the PC/PCC significantly decreases with increased plasma glucose levels in young, healthy individuals [21] as well as in cognitively normal elderly individuals [22–24]. The reduction in PC/PCC neuronal activity has been shown to occur in cognitively normal individuals with plasma glucose levels between 100 and 110 mg/dL [25] as well as in individuals developing insulin resistance [26]. Reversibly increasing and decreasing plasma glucose levels decease and increase PC/PCC neuronal activity, respectively, in cognitively normal individuals with T2DM [27]. Cerebral blood flow can also decrease in the PC/PCC as plasma glucose levels increase [21]. More recently, we measured net glucose metabolism using ^18^F-FDG PET with arterial blood sampling in young, healthy individuals under fasting and glucose loading conditions, and confirmed that glucose loading can reduce glucose metabolism (i.e., neuronal activity), especially in the PC/PCC [28]. These findings prompt the question of how increased plasma glucose levels decrease neuronal activity, especially in the PC/PCC.

Given the association among the DMN, AD, and T2DM, decreased functional connectivity of the DMN may be responsible for reduced neuronal activity in the PC/PCC. Therefore, we hypothesized that increased plasma glucose levels decrease the functional connectivity of the DMN even in healthy individuals without T2DM and insulin resistance, possibly thus reducing PC/PCC neuronal activity. To test this hypothesis, we used resting-state fMRI to compare the functional connectivity of the DMN in young, healthy participants under fasting and glucose loading conditions.

## Methods

### Research participants

 The study was conducted in accordance with the tenets of the Declaration of Helsinki, and was approved by the Ethics Committee of the Tokyo Metropolitan Institute of Gerontology. After a detailed explanation of the study, each participant provided written informed consent. The study was composed of 12 young, healthy participants [six males and six females, age: 30.3 ± 4.6 years (mean ± SD), range: 24–36 years]. None of the participants had a history of T2DM, and all were certified to be healthy based on the results of physical and neurological examinations, medical interviews with a neurologist, and MRI findings.

### Study protocol

Each participant visited the Tokyo Metropolitan Institute of Gerontology twice to undergo a resting-state fMRI under each of two different conditions: fasting and glucose loading. The order in which the participants presented for imaging under the two conditions was randomized. Half of the male and half of the female participants underwent the first and second resting-state fMRI sessions under fasting and glucose loading conditions, respectively. The other participants underwent the two resting-state fMRI sessions in the reverse order. The time interval between the two visits was less than 30 days. In the fasting condition, each participant visited the institute to undergo a resting-state fMRI after fasting overnight for at least 8 h. In the glucose loading condition, each participant visited the institute without having been under any dietary restriction, and was administered 75 g of glucose orally (TRELAN-G75; AY Pharma, Tokyo, Japan) approximately 30 min prior to the resting-state fMRI.

The plasma glucose levels, plasma insulin levels, and HbA1c values were measured after each resting-state fMRI, using ultraviolet absorption spectrophotometry, chemiluminescent enzyme immunoassay, and latex agglutination, respectively (SRL, Tokyo, Japan). The homeostasis model assessment of insulin resistance (HOMA-IR) was calculated as an index of insulin resistance using the following formula: HOMA-IR = (fasting glucose (mmol/L) × fasting insulin (μU/mL))/22.5.

### Magnetic resonance data acquisition

Imaging data were acquired on a Discovery MR 750w 3.0-T scanner (GE Healthcare, Milwaukee, WI) at the Tokyo Metropolitan Institute of Gerontology. High-resolution anatomical data were collected using an SPGR sequence (repetition time = 7.648 ms, echo time = 3.092 ms, flip angle = 11°, matrix size = 196 × 256 × 256, voxel size = 1.2 mm × 1.0547 mm × 1.0547 mm). Whole-brain resting-state fMRI data were collected using an echo planar imaging (EPI) sequence (repetition time = 2500 ms, echo time = 30 ms, flip angle = 73°, slice thickness = 4 mm, matrix size = 64 × 64 × 41, FOV = 192 mm × 192 mm). The participants were instructed to rest quietly with their eyes open and to avoid specific thoughts during the resting-state fMRI sessions. Subsequently, the procedure was manually reviewed to verify that all participants followed the instructions correctly.

### Resting-state fMRI data processing and independent component analysis (ICA)

The resting-state fMRI data were processed using the FMRIB Software Library version 5.0.9 (FSL; Oxford, UK) [29–31]. The first 10 volumes (images) were discarded to avoid transient signal changes before magnetization reached a steady state and to allow the participants to become accustomed to the fMRI scanning noise [32]. Then, the following 120 volumes, equivalent to 5 min of resting-state fMRI, were realigned to compensate for motion. Each motion-corrected EPI image was registered to the corresponding high-resolution SPGR image, and transformed into the Montreal Neurological Institute space using a 12-parameter affine transformation and a nonlinear transformation. The data were skull-stripped and spatially smoothed using a 5-mm full width at a half maximum Gaussian kernel, and a high-pass temporal filter of 100 s was applied.

**Fig. 1:**
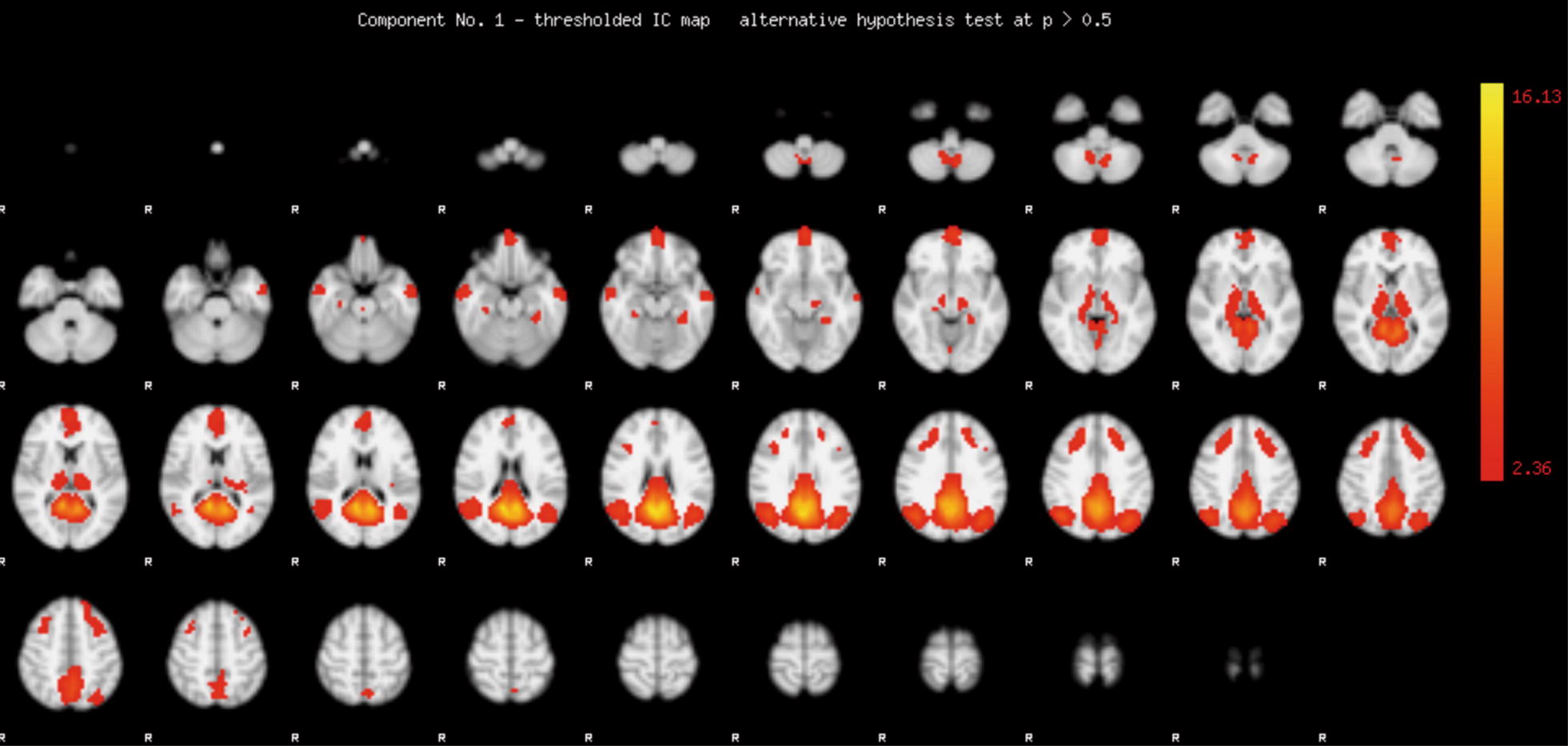
Independent component map representing the default mode network. Independent component analysis was performed on all echo planar imaging sequence images using a multi-session temporal concatenation approach implemented in FSL Melodic. The mixture model probability map was transformed into a *Z* map. The red-yellow scale represents the magnitude of *Z* values ranging from 2.36 to 16.13

Probabilistic independent component analysis (ICA) was then performed to identify the functional anatomy of the DMN, and to create a DMN mask for the subsequent seed-based analysis. A multi-session temporal concatenation approach was applied to all echo planar imaging sequence images. This approach allowed for a single 2D ICA run on the concatenated data matrix to be obtained by stacking the 2D data matrices of every data set on top of each other (https://fsl.fmrib.ox.ac.uk/fsl/fslwiki/MELODIC). FSL Melodic was used to carry out inference on the estimated maps using a mixture model and an alternative hypothesis testing approach. A threshold level of 0.5 was applied to each mixture model probability map. This threshold level implies that a voxel “survives” thresholding as soon as the probability of being in the “active” class exceeds that of being in the “background noise” class, and assumes that the probability of false-negative and false-positive findings is equal [33, 34]. Of the 25 IC maps created by FSL Melodic, we identified one IC map representing the default mode network (Fig. 1).

### Seed-based analysis and statistical analysis

**Fig. 2:**
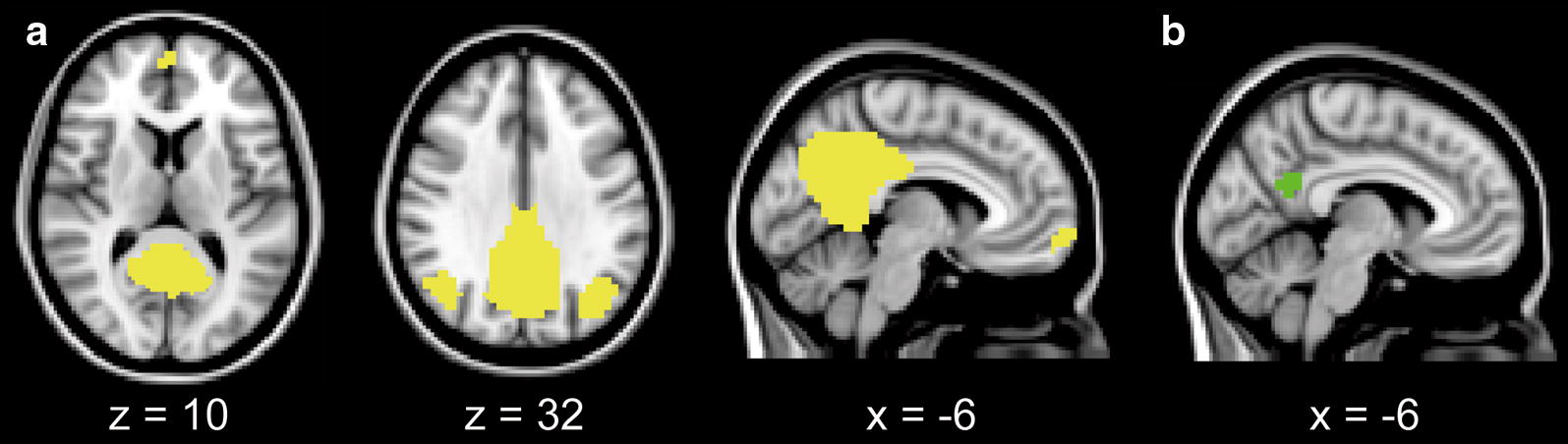
Masks for the representative components of the DMN (**a**) and PC/PCC (**b**) in the Montreal Neurological Institute space. The representative components of the DMN were extracted from the IC map shown in Fig. 1, and used as a mask for the DMN (**a** yellow). The voxels with the highest statistical values were extracted from the IC map shown in Fig. 1, and used as a mask for the PC/PCC (**b** green). The mask volume for the PC/PCC was 2360 mm^3^. *DMN* default mode network, *IC* independent component, *PC/PCC* precuneus/posterior cingulate cortex

The thresholded IC map, shown in Fig. 1, included the representative components of the DMN: the PC/PCC, the MPFC, and the LPTC. These components were extracted from the IC map and used as a mask for the DMN (Fig. 2a). Using the mask for the DMN as a seed, the mean time series across all voxels within the seed was extracted from each EPI image. A first-level analysis was performed for each 4D EPI image. The extracted mean time series was set as a covariate. We added the following variables as nuisance regressors: mean signals of cerebrospinal fluid and white matter, and metrics of motion-related artifact created by FSL Mcflirt and Motion Outliers [35, 36]. A one-sample *t* test was then performed as a higher-level analysis for each of the two conditions to assess the within-group functional connectivity of the DMN, using FSL Feat (https://fsl.fmrib.ox.ac.uk/fsl/fslwiki/FEAT). *Z* statistic images were thresholded using clusters determined by *Z* > 2.3 and a corrected cluster significance of *P* < 0.05.

A between-group analysis was then performed to test the hypothesis that increased plasma glucose levels decrease the functional connectivity of the DMN. The central area of the PC/PCC was extracted from the IC map as shown in Fig. 1 and used as a mask for the PC/PCC (Fig. 2b). The mask was moved on each *Z* map that was created in the first-level analysis, as described above. The individual mean *Z* value within the mask was calculated, and used as the index of the magnitude of the functional connectivity of the DMN in the PC/PCC. To assess the effects of glucose loading on the functional connectivity of the DMN in the PC/PCC, *Z* values were compared between the fasting and glucose loading conditions using a one-tailed Wilcoxon signed-rank test. The null hypothesis was that the functional connectivity of the DMN in the glucose loading condition was not lower than in the fasting condition. Additionally, in order to assess whether any factors affected the changes in the functional connectivity of the DMN after glucose loading, a multiple regression analysis was employed using the difference in *Z* values between the two conditions as a dependent factor and the order of conditions, gender, HOMA-IR, fasting plasma glucose and insulin levels, and HbA1c values as independent factors. Statistical significance was set at *P* < 0.05. All statistical analyses were conducted using SPSS Statistics version 22 (IBM, Armonk, NY).

## Results

The demographic characteristics are presented in Table 1. After glucose loading, plasma glucose and insulin levels significantly increased (glucose: *Z* = 2.158, *P* = 0.031, insulin: *Z* = 3.061, *P* = 0.002, two-tailed Wilcoxon signed-rank test). All participants were confirmed to be free of T2DM and insulin resistance on the basis of HOMA-IR, fasting plasma glucose levels, and HbA1c values [37].

**Table 1:** Demographic and clinical characteristics

Subject	Age	Sex	HbA1c (%)	Fasting			Glucose loading	
				Glucose (mg/dL)	Insulin (μU/mL)	HOMA-IR	Glucose (mg/dL)	Insulin (μU/mL)
1	34	M	5.2	94	2.7	0.62	126	37.8
2	34	M	5.8	80	6.5	1.28	164	32.2
3	27	M	5.5	84	0.8	0.16	85	4.8
4	32	F	4.7	90	4.4	0.98	96	18.1
5	30	F	5.1	82	3.4	0.69	100	27.8
6	36	F	5.2	87	2.7	0.58	117	33.4
7	23	M	5.4	91	2.4	0.53	115	18.7
8	26	F	4.9	94	4.2	0.98	84	8.0
9	37	F	5.1	87	1.8	0.40	186	87.4
10	32	M	5.1	90	3.4	0.75	123	29.1
11	24	M	4.9	89	1.9	0.42	83	29.0
12	29	F	5.3	82	4.3	0.88	75	26.5
Mean			5.2	87.5	3.2	0.69	112.8	29.4

*HOMA-IR* homeostasis model assessment of insulin resistance

**Fig. 3:**
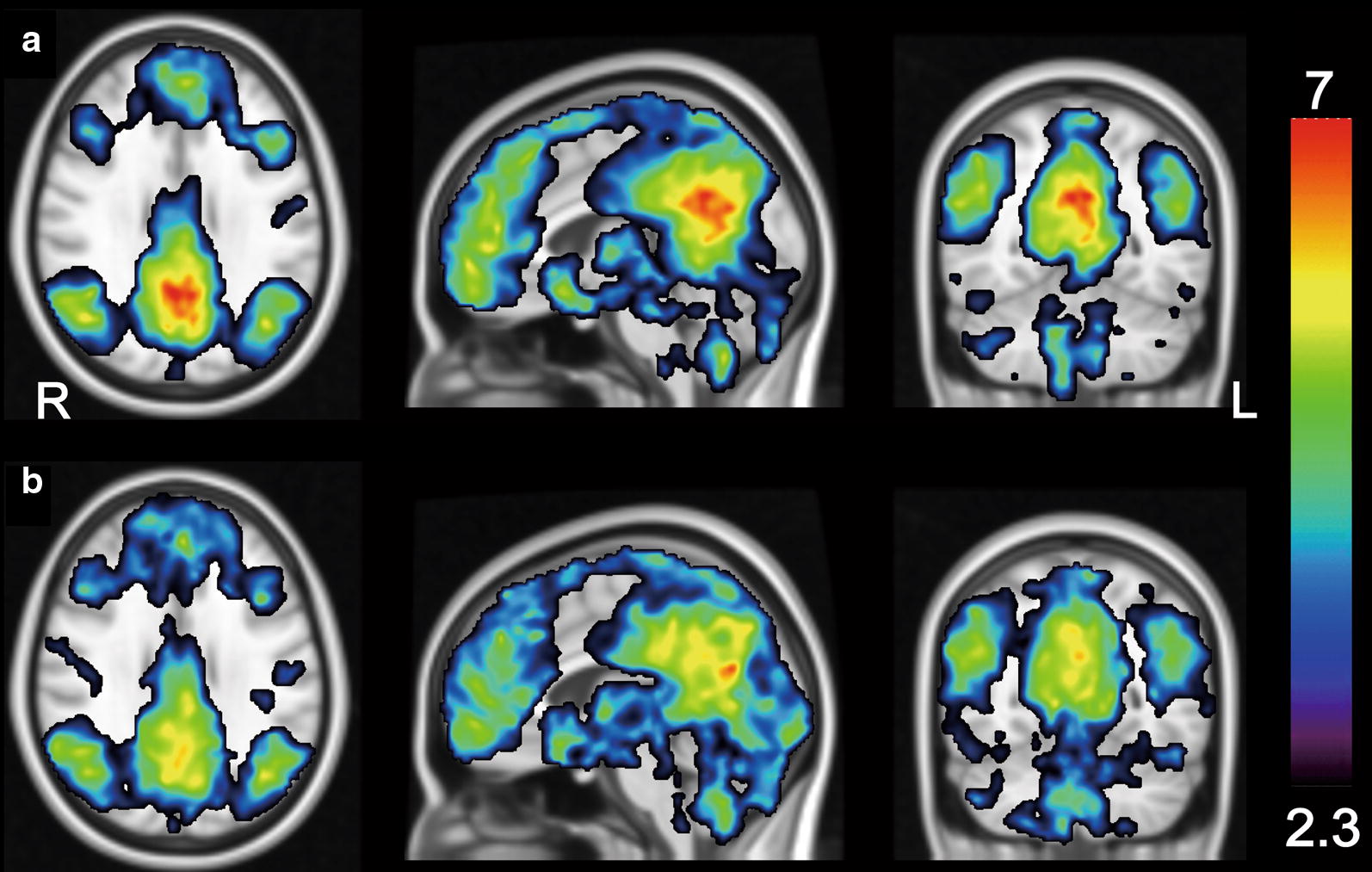
Within-group functional connectivity of the DMN using a one-sample *t* test. A seed was placed on the representative components of the DMN as shown in Fig. 2a. The magnitude of the DMN functional connectivity is displayed in the fasting condition (**a**) and glucose loading condition (**b**). The threshold was set at *Z* > 2.3 and cluster-corrected *P* < 0.05. The rainbow scale represents the magnitude of the *Z* values. *R* right, *L* left, *DMN* default mode network

**Fig. 4:**
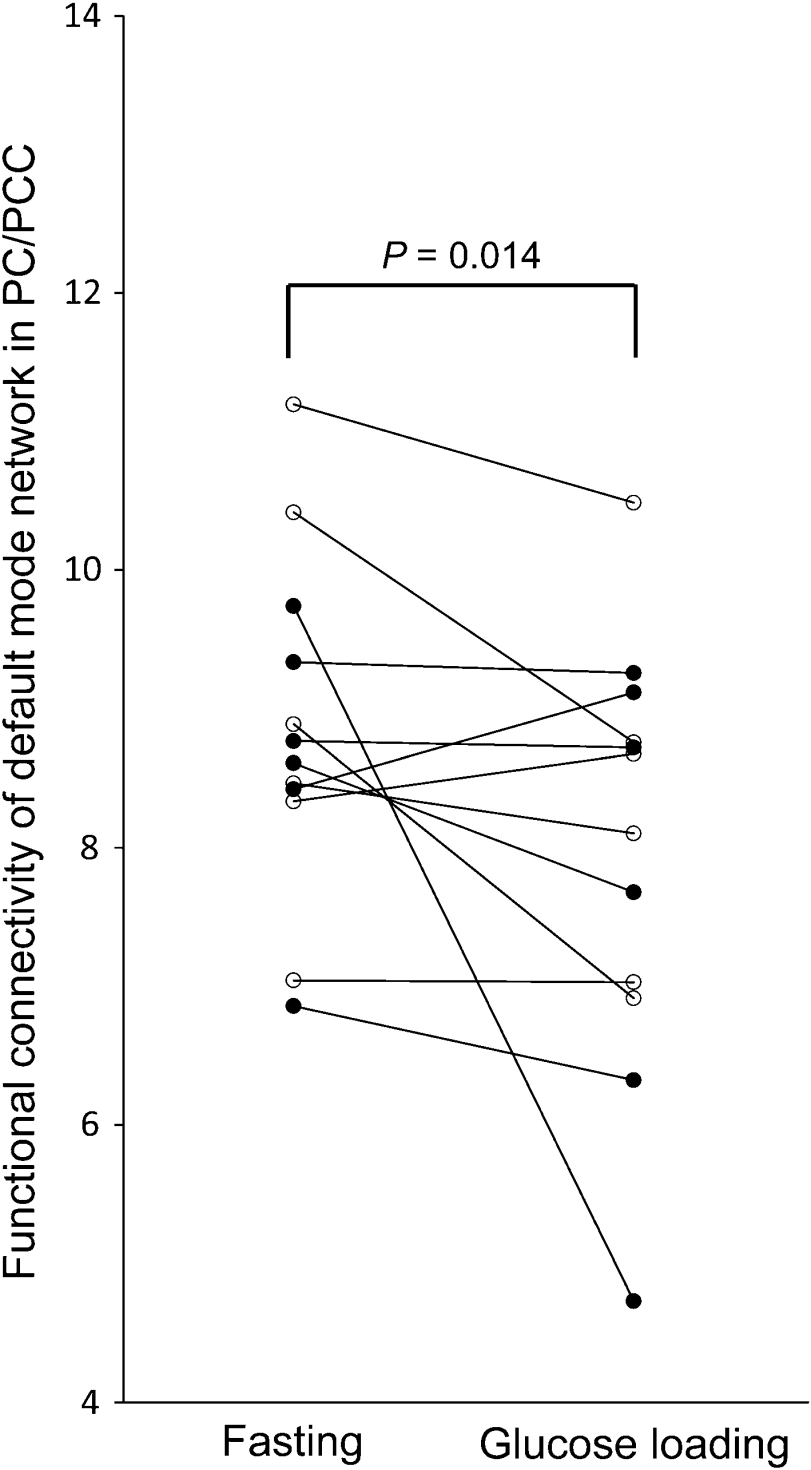
Differences in the functional connectivity of the DMN in the PC/PCC. The y-axis represents the mean *Z* values in the PC/PCC shown in Fig. 3, which was used for the index of the magnitude of the functional connectivity of the DMN in the PC/PCC. The functional connectivity was significantly lower in the glucose loading condition compared with the fasting condition (*Z* = 2.197, *P* = 0.014, one-tailed Wilcoxon signed-rank test). Closed and open circles represent males and females, respectively. *DMN* default mode network, *PC/PCC* precuneus/posterior cingulate cortex

The results of the one-sample *t* tests (*Z* > 2.3, cluster-corrected *P* < 0.05) are shown in Fig. 3. The representative components of the DMN (PC/PCC, MPFC, and LPTC) were detected in the two conditions. The results of the between-group analysis of the magnitude of the DMN functional connectivity in the PC/PCC are shown in Fig. 4. The functional connectivity of the DMN in the PC/PCC was significantly lower in the glucose loading condition than in the fasting condition (*Z* = 2.197, *P* = 0.014, one-tailed Wilcoxon signed-rank test).

Multiple regression analyses revealed no significant factors that may have affected the changes in functional connectivity between the two conditions [*R*^2^ = 0.176, *F*(6, 5) = 0.178, *P* = 0.971, order of conditions: *t* = 0.380, *P* = 0.719, gender: *t* = 0.362, *P* = 0.732, HOMA-IR: *t* = 0.041, *P* = 0.969, fasting plasma glucose: *t* = 0.310, *P* = 0.769, fasting plasma insulin: *t* = 0.038, *P* = 0.971, HbA1c: *t* = 0.721, *P* = 0.503].

## Discussion

The primary objective of this study was to investigate the effects of glucose loading on the functional connectivity of the DMN in young, healthy subjects free of T2DM and insulin resistance, using resting-state fMRI. The functional connectivity of the DMN is known to decrease in patients with T2DM, characterized by insulin resistance and increased plasma glucose levels [11–13]. To the best of our knowledge, this is the first study showing that after glucose loading, the functional connectivity of the DMN is decreased even in healthy individuals without T2DM and insulin resistance. Zhang and colleagues recently evaluated the acute effects of insulin administration on the resting-state brain network in patients with T2DM, and showed that insulin administration increased the functional connectivity between the hippocampus and the DMN [38]. Because insulin administration induces a reduction in plasma glucose levels, their findings could be restated as showing that a decrease in plasma glucose levels increases the functional connectivity of the DMN. Thus, their findings from patients with T2DM are consistent with our results. However, because the number of participants was relatively small in the present study, our results require further validation in a future study with a large number of participants.

One of the concerns of this study is a lack of understanding as to what the reduction in the functional connectivity of the DMN by glucose loading physiologically reflects. There are several studies using ^18^F-FDG PET, reporting that increased plasma glucose levels decrease glucose metabolism (i.e., neuronal activity), especially in the PC/PCC [24, 28]. In a dynamic ^18^F-FDG PET study with arterial blood sampling, which directly measured net glucose metabolism, glucose loading decreased glucose metabolism in DMN-related regions, especially in the PC/PCC, in young, healthy individuals free of T2DM and insulin resistance [28]. Considering these findings, reduced functional connectivity of the DMN by glucose loading is possibly responsible for reduced neuronal activity in DMN-related regions, especially in the PC/PCC.

Interestingly, plasma glucose levels in the prediabetes range of 100–126 mg/dL [39] are associated with cognitive decline, as measured using a battery of neuropsychological tests [40–42]. There is an inverse association between plasma glucose levels and Mini Mental State Examination scores in individuals at high risk for cardiovascular disease [43]. In a sample of non-T2DM elderly subjects, individuals with higher plasma glucose levels tended to have lower Mini Mental State Examination scores [40]. A longitudinal study with a median follow-up of 6.8 years showed that higher glucose levels might be related to an increased risk for dementia, even among individuals without T2DM [44]. Although it remains unclear as to why mildly increased plasma glucose levels induce cognitive decline, the phenomenon may be speculated as follows: increased plasma glucose levels reduce the functional connectivity of the DMN as well as neuronal activity in its components, particularly the PC/PCC, which is a central core for regulating complex cognition and behavior [45, 46]. As a result, subclinical cognitive decline may occur even in individuals without T2DM. This speculation may be important to explain the functional link between T2DM and AD, although future studies are needed to elucidate this hypothesis.

In summary, glucose loading can reduce the DMN functional connectivity and PC/PCC neuronal activity in healthy participants. Although the mechanism underlying this phenomenon is unclear, cholinergic and glutamatergic neurotransmitter systems may play an important role in modulating the functional connectivity of the DMN and neuronal activity in the PC/PCC. This is because both the DMN and PC/PCC are anatomically crucial in regulating complex cognition and behavior [4–6, 45, 46], and cholinergic and glutamatergic systems are associated with cognitive function [47]. Moreover, cholinergic enhancement is reported to increase neuronal activity in the PC/PCC [48]. Hence, glucose loading may modulate these neurotransmitter systems, possibly reducing the functional connectivity of the DMN and neuronal activity in the PC/PCC. However, further investigation is needed to elucidate this speculation.

## Conclusions

The present study showed that glucose loading reduces the functional connectivity of the DMN in the PC/PCC in young, healthy participants free of T2DM and insulin resistance. Taken together with the previous knowledge that glucose loading decreases neuronal activity in the PC/PCC, the present results suggest that decreased functional connectivity of the DMN is possibly responsible for reduced PC/PCC neuronal activity in healthy individuals with increased plasma glucose levels.

## Abbreviations

DMN: default mode network
AD: Alzheimer’s disease
T2DM: type 2 diabetes mellitus
PC/PCC: precuneus/posterior cingulate cortex
MPFC: medial prefrontal cortex
LPTC: lateral parietotemporal cortex
^18^F-FDG: fluorine-18-labeled fluorodeoxyglucose
PET: positron emission tomography
fMRI: functional magnetic resonance imaging
HOMA-IR: homeostasis model assessment of insulin resistance
